# Electrospun Aligned Fibrous Arrays and Twisted Ropes: Fabrication, Mechanical and Electrical Properties, and Application in Strain Sensors

**DOI:** 10.1186/s11671-015-1184-9

**Published:** 2015-12-09

**Authors:** Jie Zheng, Xu Yan, Meng-Meng Li, Gui-Feng Yu, Hong-Di Zhang, Wojciech Pisula, Xiao-Xiao He, Jean-Luc Duvail, Yun-Ze Long

**Affiliations:** College of Physics, Qingdao University, Qingdao, 266071 China; Max-Planck-Institute for Polymer Research, Ackermannweg 10, 55128 Mainz, Germany; Institut des Matériaux Jean Rouxel, CNRS, Université de Nantes, Nantes, France; Collaborative Innovation Center for Marine Biomass Fibers, Materials and Textiles of Shandong Province, Qingdao University, Qingdao, 266071 China

**Keywords:** Electrospinning, Aligned arrays, Twisted ropes, Electrical properties, Strain sensors

## Abstract

Electrospinning (e-spinning) is a versatile technique to fabricate ultrathin fibers from a rich variety of functional materials. In this paper, a modified e-spinning setup with two-frame collector is proposed for the fabrication of highly aligned arrays of polystyrene (PS) and polyvinylidene fluoride (PVDF) nanofibers, as well as PVDF/carbon nanotube (PVDF/CNT) composite fibers. Especially, it is capable of producing fibrous arrays with excellent orientation over a large area (more than 14 cm × 12 cm). The as-spun fibers are suspended and can be easily transferred to other rigid or flexible substrates. Based on the aligned fibrous arrays, twisted long ropes are also prepared. Compared with the aligned arrays, twisted PVDF/CNT fiber ropes show enhanced mechanical and electrical properties and have potential application in microscale strain sensors.

## Background

Electrospinning (e-spinning) is a simple and versatile technique to fabricate fibers with diameters typically ranging from a few micrometers down to 10 nm or less. In a traditional e-spinning process, a charged jet is ejected from a Taylor cone and rushes onto the grounded collector under the driving force of an electric field. After solvent evaporation, solid fibers with uniform diameter are randomly deposited on the collector [1–5]. In the past decade, numerous ultrathin fibers originated from polymer, metal, ceramic, and glass have been prepared by e-spinning, and their potential applications in optoelectronics, sensors, catalysis, textiles, filters, fiber reinforcement, tissue engineering, drug delivery, and wound healing have also been extensively explored [1, 6–9].

Normally, the products fabricated by traditional e-spinning are randomly oriented fibers known as a nonwoven mat. In order to extend the potential applications of e-spinning, a lot of modified e-spinning techniques have been proposed to obtain fibers with desired morphologies such as aligned fibrous arrays and twisted ropes. For example, aligned fibers can be prepared by introducing a gap into the conventional collector [10], adding an auxiliary electric or magnetic field [11], double spinning, [12] near-field e-spinning [13–18], and rotating collector [19]. Twisted fiber bundles have also been fabricated by a few means such as dual collection rings [20], AC e-spinning [21], a modified setup with two collectors [22], or with an alternating electric field [23]. The twisted nanofiber rope is promising in the applications of artificial muscle, electron devices, and suture materials [20]. Nevertheless, there are few methods by which both well-aligned fibers and twisted ropes can be fabricated.

So far, the applications of electrospun fibers have been paid much attention due to their unique physical, chemical, and even biological properties, especially in the field of electrical sensors. For instance, piezoelectric materials such as ZnO and polyvinylidene fluoride (PVDF) have been electrospun into aligned fibers and then integrated into a nanogenerator. If this device is pressed or bended by a strain, the current will be induced, resulting from the piezoelectric properties of the functional fibers [24, 25]. Lotus et al. modified the traditional e-spinning setup by introducing a rotating and a stationary collector [26], and obtained semiconducting twisted ZnO/NiO composite yarns, exhibiting a rectifying behavior of a p-n junction [27]. In addition, twisted microropes of poly(3,4-ethylenedioxythiophene):poly(styrene sulfonate)-polyvinyl pyrrolidone (PEDOT:PSS-PVP) fibers doped with ionic liquid showed a linear correlation of electrical conductivity with a strain up to 35 % and a repeatable cycle loop of tensile-resilience [28].

Moreover, efforts have been done to study a nanocomposite made of PVDF and carbon nanotube (CNT) and the interfacial interactions between the two components [29–31]. For example, the difference of the Raman spectra of CNT and CNT dispersed in the PVDF was ascribed to the interfacial interaction between the fluorine of PVDF and the CNTs [29]. The interfacial interaction between the single-walled carbon nanotube (SWCNT) and PVDF and the extensional force experienced by the nanofibers in the e-spinning and collection processes could work synergistically to induce highly oriented β-form crystallites of PVDF extensively [30]. Although the morphological and structural properties and polymorphic behaviors as well as interfacial interactions between PVDF and embedded CNT have been studied, the electrical and mechanical properties of electrospun PVDF/CNT fibers have not been reported intensively.

In this letter, we report on a novel e-spinning device with a set of a modified frame collector, by which highly ordered arrays of polystyrene (PS) and PVDF nanofibers, as well as PVDF/multiwall CNT (MWCNT) composite nanofibers, can be fabricated. It is worthy to note that the spun fibrous arrays show excellent orientation over a large area (more than 14 × 12 cm^2^). Moreover, twisted ropes can be also prepared successfully with ~10 cm in length by the same technique. The twisted PVDF/CNT composite ropes exhibit improved mechanical performance and conductivity compared with aligned fibrous arrays. In addition, an electrical device has been integrated with high-strain sensitivity based on the twisted rope, indicating good potential in the high-strain sensor.

## Methods

### Apparatus

**Fig. 1:**
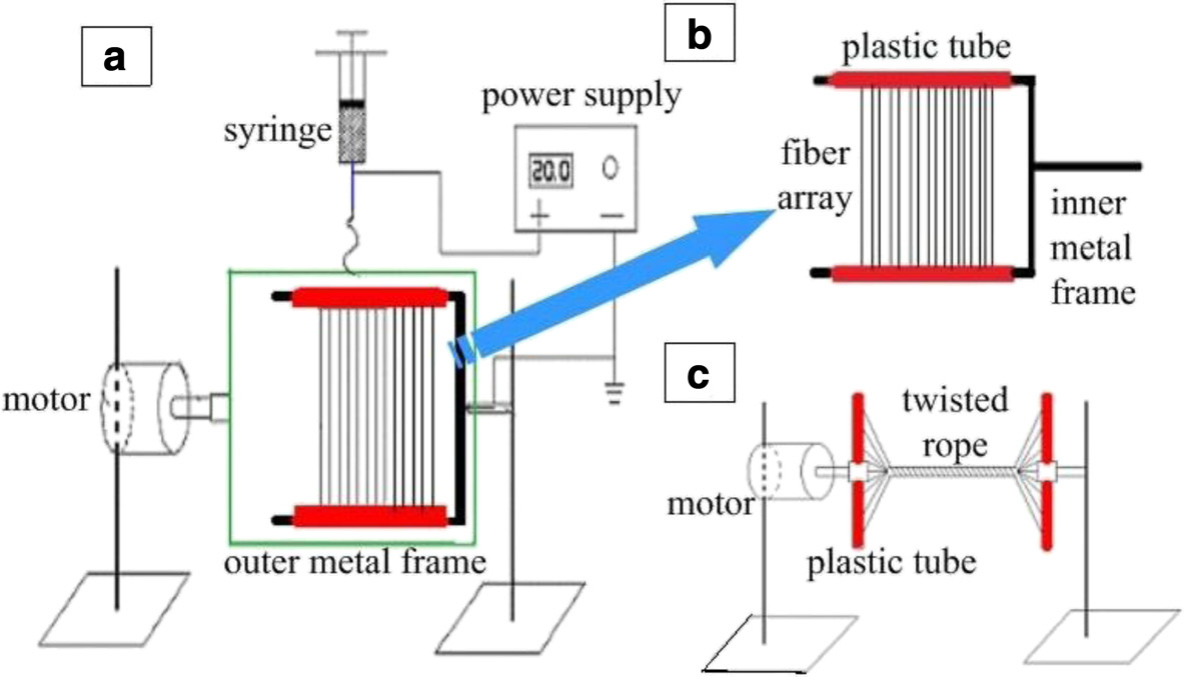
Schematic illustration of the modified e-spinning setup

The illustration of the setup used in this work is shown in Fig. 1(a). Compared with the traditional e-spinning, this setup has a two-frame collector that consists of an inner one (U-shape) and an outer one (rectangle). For the inner frame (14 × 12 cm^2^), two parallel sides are both covered with thin plastic tubes, as shown in Fig. 1(b), while the outer one (18 × 16 cm^2^) is connected with a motor. During the e-spinning process, the inner frame is stationary but the outer one is rotated with the motor. Here, it is worthy to note that the plastic tubes can be removed freely. Electrospun fibers are deposited and stretched across two parallel plastic tubes to form highly aligned fibrous arrays. The pair of plastic tubes with aligned fibers can be removed from the inner frame and transferred to the rotating part of the setup. As shown in Fig. 1(c), one tube is rotated with the motor and another is fixed to the iron support. Subsequently, a twisted rope can be obtained by rotating the tube connected with the motor. In order to get twisted ropes with a longer size and higher quality, both plastic tubes can be compressed along their axis to improve the density of resultant fibers. Compared with the e-spinning setup with a one-frame collector, the main role of the outer frame here is to obtain aligned fiber array and that of the inner frame is to collect better aligned fibers and transfer the fiber array from the collector.

### Preparation of Spun Solution and Electrospinning

Polystyrene (PS) solution was prepared by dissolving 2.0 g PS (average molecular weight of 250,000, ACROS) in 8.0 g tetrahydrofuran (THF). After being stirred for 4 h at room temperature, the PS solution was kept at room temperature for 0.5 h before e-spinning. The PVDF solution was prepared by a similar way by dissolving 1.25 g PVDF (average molecular weight of 275,000, Aldrich) in the mixture of 2.5 g acetone and 2.5 g dimethylformamide (DMF) with 2 h stirring at 60 °C. CNT precursor solution was prepared by dissolving 1.2 g CNTs (Chengdu Organic Chemical Co., LTD) in the mixture of 16.8 g acetone and 2.0 g CNT dispersion (TNWIDS, Chengdu Organic Chemical Co., LTD). PVDF/CNT solution was prepared by dissolving CNT precursor solution and PVDF in the mixture of acetone and DMF (the weight ratio is 1:1). Here, the contents of the CNTs in the final fibers were 8.0, 12.4, and 16.7 wt%, respectively. The PVDF/CNT solution was stirred for 4 h in a water bath at 60 °C before e-spinning.

A high-voltage DC power supply (Tianjin Dongwen, China) was employed to generate voltages. The spun solution was loaded into a 5.0-ml syringe with a stainless spinneret (inner diameter 0.72 mm) which is connected with the anode of the power supply. The applied spinning voltage was 20 kV, and the vertical distance (work distance) between the spinneret and the top of the grounded frame was 8 cm, while the inner frame was placed horizontally at the beginning. The feed rate (e.g., 0.5 ml min^−1^) could be controlled by a syringe pump. During the electrospinning (ES) process, the rotating speed of the outer frame was set to 600 rpm. All experiments were carried out at room temperature.

### Characterization

A digital camera, an optical microscope (SMZ-168), and a scanning electron microscope (SEM; JEOL JSM-6390) were used to observe morphologies of the ES fibers. All samples are coated with an evaporated gold thin film before SEM imaging to ensure higher conductivity. The PVDF/CNT fibers were characterized by a transmission electron microscope (TEM; HITACHI H-9000), and Fourier transform infrared spectroscopy (FTIR) using a Thermo Scientific Nicolet iN10 spectrometer and absorbance data were processed for the wave number range 700–1000 cm^−1^. A mechanical test system (Agilent T150 UTM) and a set of an electrical measurement system (Keithley 6220 and Vitech triple output DC power supply) were used to measure the mechanical and electrical properties of the fiber bundles and ropes, separately.

## Result and Discussion

### Fabrication of Aligned Fibrous Arrays and Twisted Ropes

**Fig. 2:**
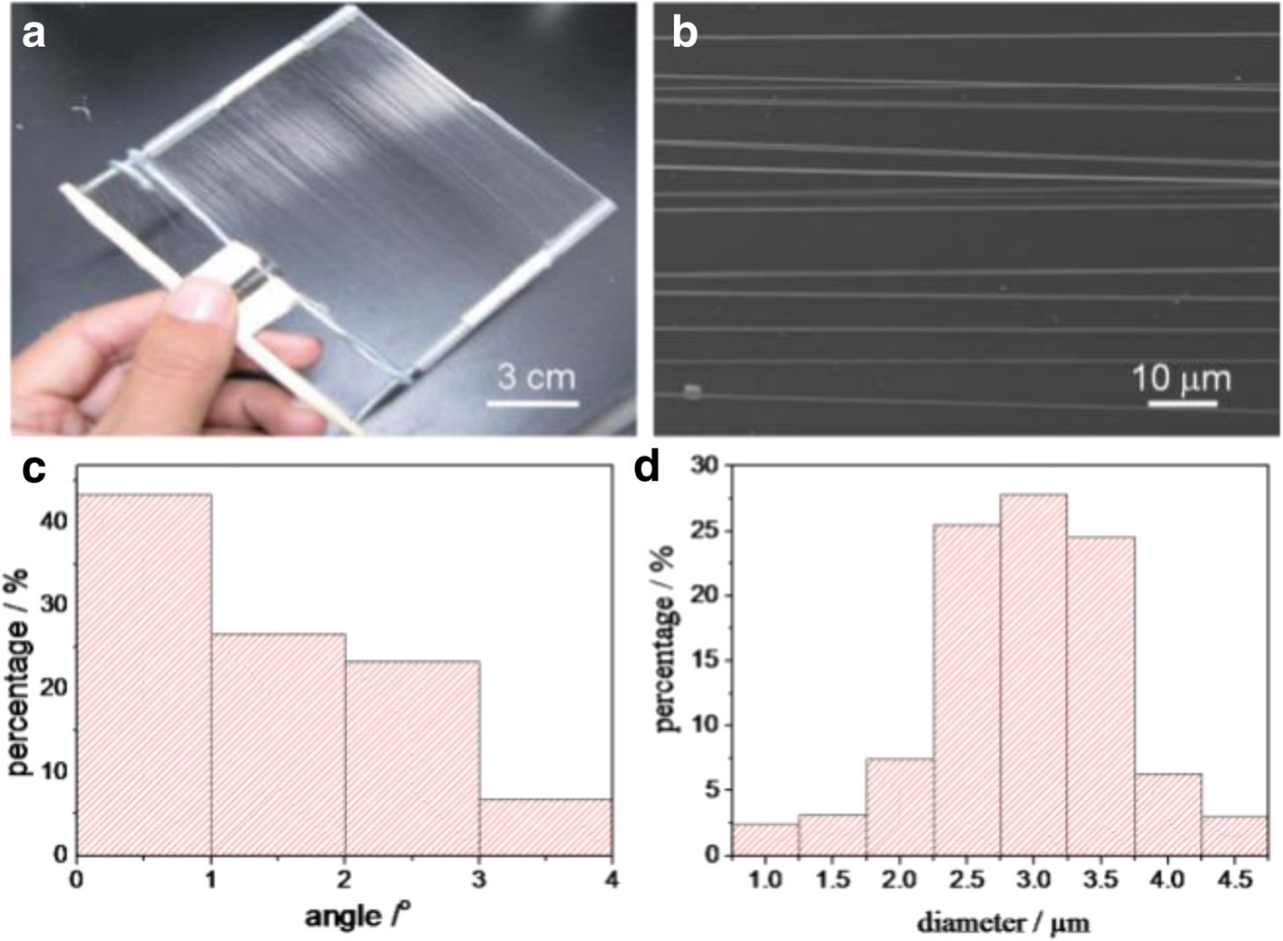
**a** Highly ordered arrays of PS fibers over a large area (more than 14 × 12 cm^2^). **b** SEM image of the aligned PS fibers from which the average diameter was determined. **c** The statistical analysis of the alignment of ordered PS fibers. **d** Diameter distribution of the aligned fibers in (**b**).

Firstly, we utilized the modified e-spinning setup to collect fibers (Fig. 1(a)). During the spinning process, fibers were attracted by and stretched across the pair of parallel plastic tubes of the inner frame, resulting in the formation of an aligned fibrous array with the orientation perpendicular to the tubes. It is worth noting that the size of the aligned array is as large as 14 × 10 cm^2^, as shown in Fig. 2a. From Fig. 2b, we can observe the excellent alignment of the PS fibers. We define the direction perpendicular to the plastic tubes as the base direction. Results of statistical analysis from more than 40 fibers indicate that the angle between the axis of fibers and base direction is within the limit of 4° for all fibers (Fig. 2c). Especially, the angle is less than 2° for about 70 % of fibers, revealing excellent fiber orientation. In addition, the diameter of the PS fibers is uniform, arranging from 1.1 to 4.5 μm with an average value of 2.89 μm, as shown in Fig. 2d.

**Fig. 3:**
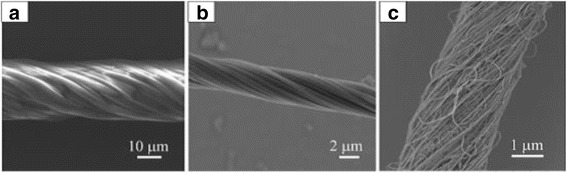
SEM images of twisted polymer fiber ropes: **a** PS, **b** PVDF, and **c** PVDF/CNT

Based on the aligned fibrous array prepared above, we fabricated twisted ropes using the setup shown in Fig. 1(c). Herein, after taking two thin plastic tubes with as-spun fibers down from the inner frame, we fixed them with the two steel supports in which one tube can be rotated with the motor and the other one remains stationary. Before rotation, we pressed two tubes along their axis in order to increase the density of the fibers. This procedure can not only further align the ES fibers but also increase the length of the final rope. With the rotation of the tube connected with the motor for 5–10 s, the aligned fibrous arrays were twisted into a rope with several tens of micrometers in diameter, as shown in Fig. 3a.

### Influence of the ES Parameters on the Morphology of Aligned Fibrous Arrays and Twisted Ropes

**Fig. 4:**
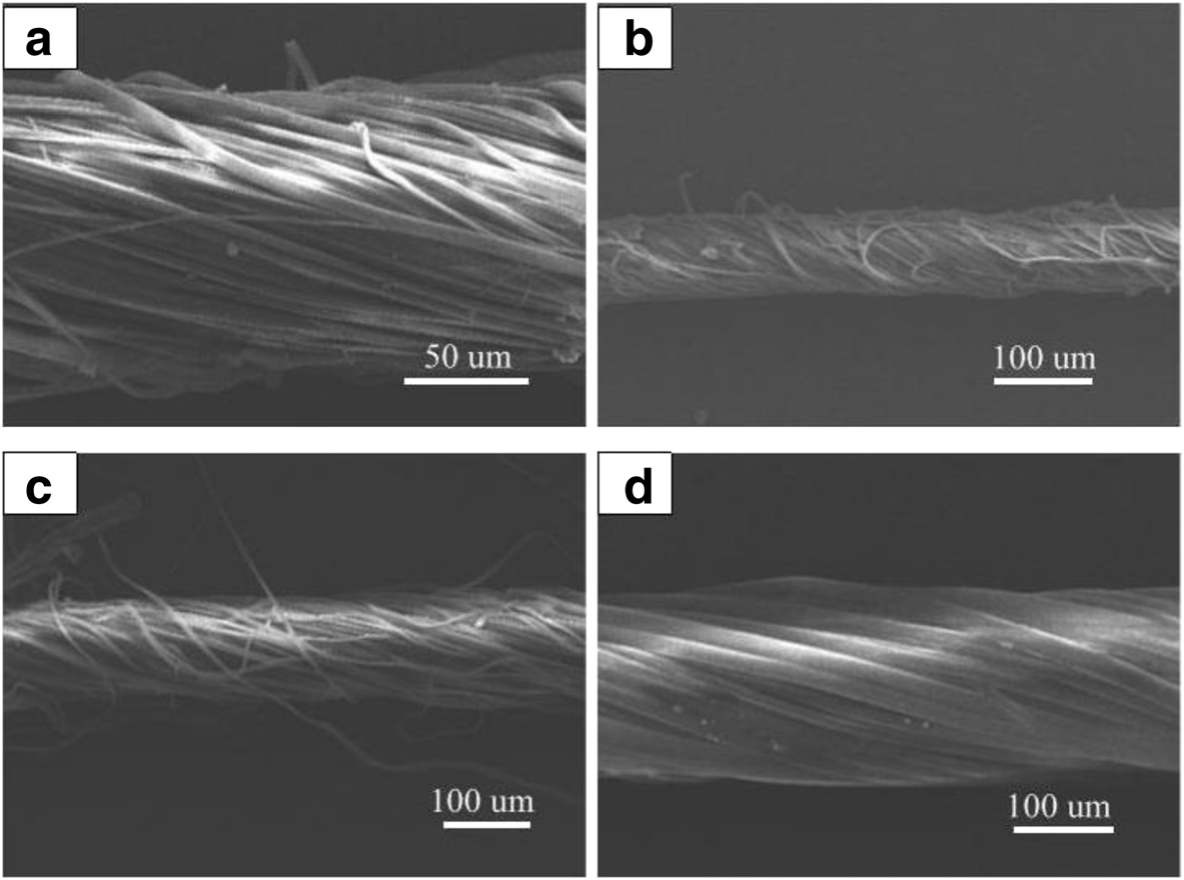
SEM images of the twisted nanofiber ropes prepared at different included angles: **a** 60°, **b** 45°, **c** 30°, and **d** 0°

In our modified ES setup, there are two collecting frames: the stationary inner frame and the rotating outer frame. Interestingly, we observe that the position of the inner frame, that is, the plane angle between inner frame and the horizon, plays a critical role in the orientation of the deposited fibers. To explore this factor, we have carried out a series of experiments as following. The inner frame with two plastic tubes on which the ES fibers were collected was set at 60°, 45°, 30°, and 0° with respect to the horizontal plane. All other experimental parameters were kept unchanged. From Fig. 4, we can see that the horizontal position of the inner frame (0°) is the best parameter for which the highest quality of the twisted rope is obtained. However, with the inner frame in other positions (60°, 45°, and 30°), a few disordered fibers are observed.

**Fig. 5:**
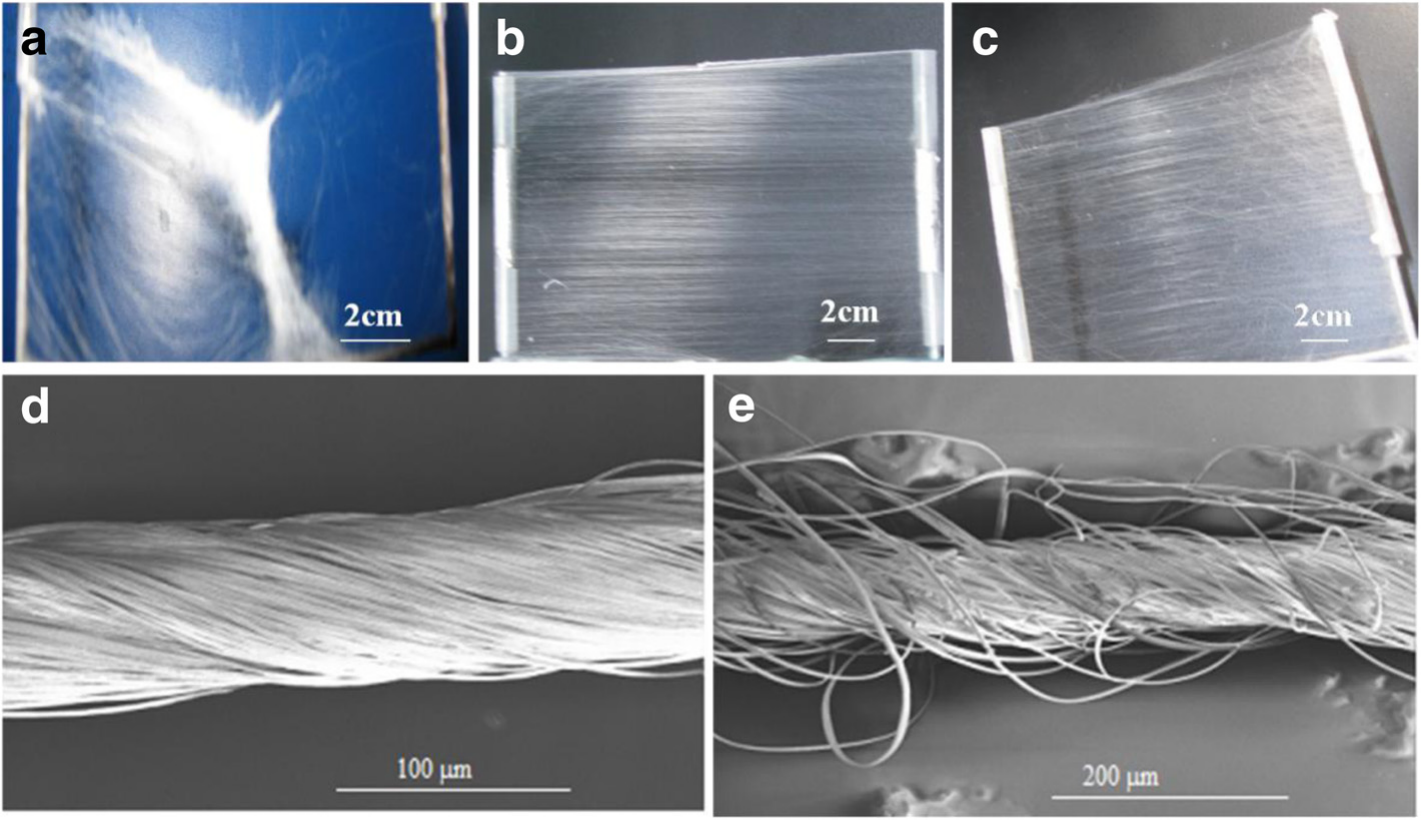
Optical images of the electrospun nanofibers obtained at different rotating speeds of the outer frame: **a** 100 rpm, **b** 600 rpm, **c** 800 rpm, **d** SEM image of the twisted nanofiber rope with smooth surface obtained from (**b**), and **e** SEM image of the twisted nanofiber rope obtained from (**c**)

On the other hand, the rotating speed of the outer frame has a great influence on the alignment of ES fibers. A set of experiments was carried out with different rotating speeds of the outer frame, and the corresponding optical images are shown in Fig. 5. Since the slow rotation (100 rpm) does not match to the spinning speed, fibrous bundles with a low orientation are formed instead of aligned arrays (Fig. 5a). Fibers with excellent orientation can be achieved using the rotation speed of 600 rpm (Fig. 5b). When the speed increases to 800 rpm, the aligned fibers are covered with a few randomly arranged ones, which reduce the quality not only of the whole oriented fiber system (Fig. 5c) but also of the subsequent twisted rope (Fig. 5d, e) to some extent.

### Fabrication of PVDF/CNT Twisted Ropes

**Fig. 6:**
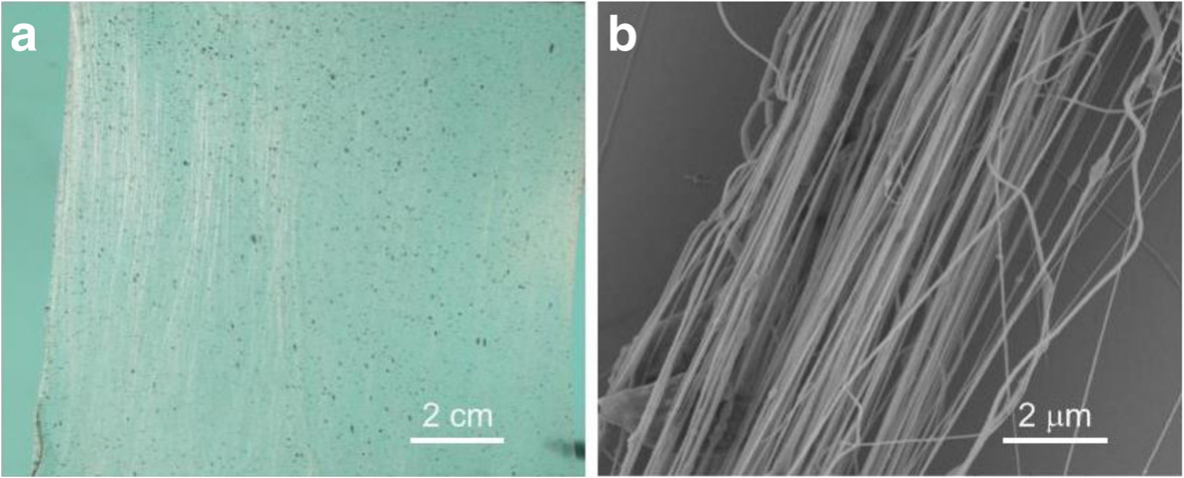
**a** Optical image of the aligned fibrous PVDF/CNT array. **b** SEM image of the PVDF/CNT bundle after compressing the aligned fiber array

CNTs have attracted much attention due to their outstanding structural, electrical, thermal, mechanical, and chemical performances [32–34]. Various composite materials containing CNTs have superior mechanical and electrical properties [35–40]. To increase the mechanical and electrical properties of the twisted ropes, the PVDF/MWCNT composite fibers were fabricated by using our modified setup (the applied voltage was 20 kV, the work distance was 8 cm, and the spinning time was the same as PS fibers). The aligned fibrous array consists of PVDF/CNT composite fibers with the area up to 14 × 12 cm^2^ as evident from the optical image in Fig. 6a. As mentioned in the experimental section, we increased the density of fibers by pressing the plastic tubes along their axis in order to improve the quality of the twisted ropes. The SEM images in Fig. 6b exhibit the morphology of the fiber bundle with a higher fiber density. After twisting, a composite rope with about 2 μm in diameter is formed (Fig. 3c).

**Fig. 7:**
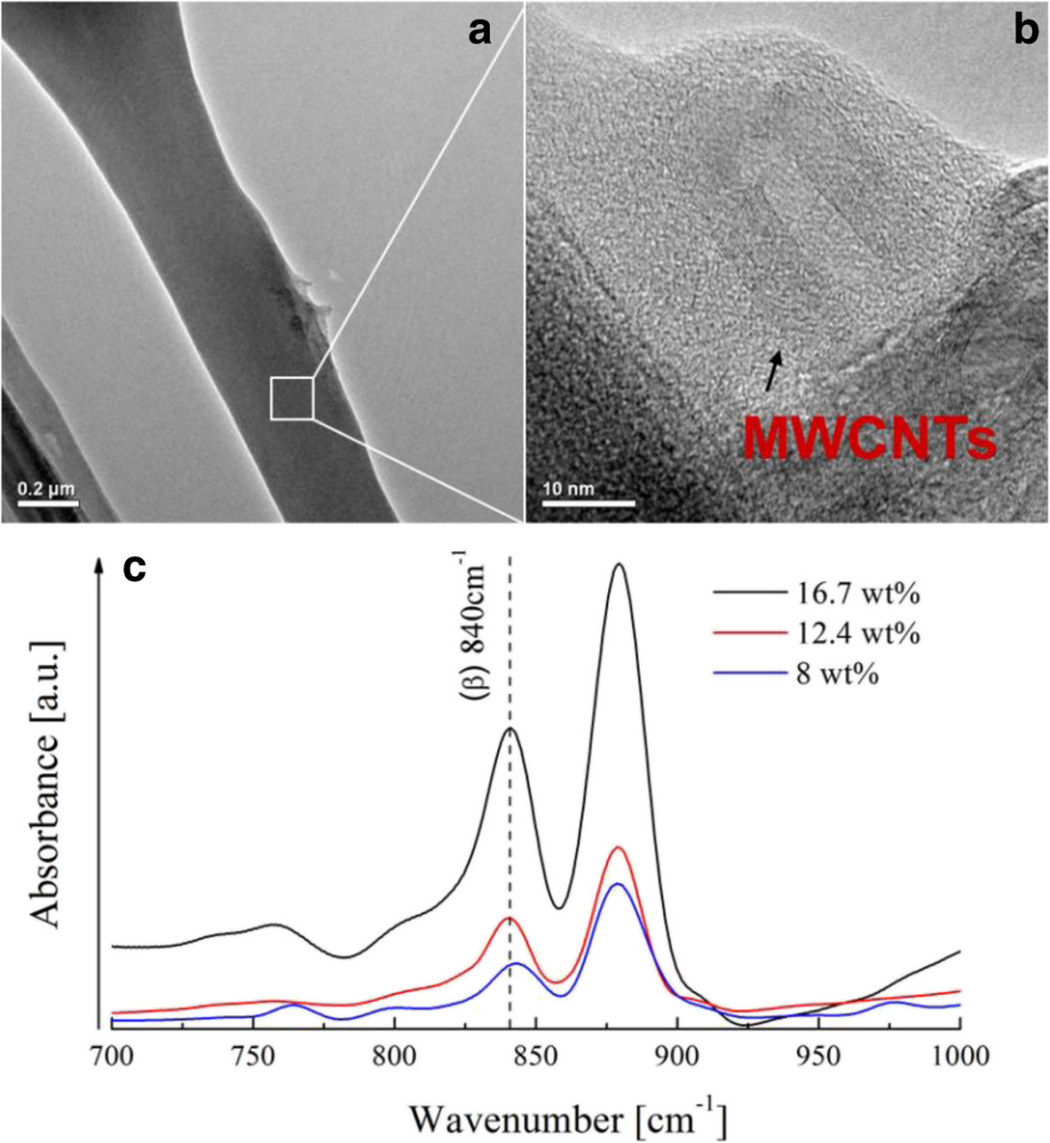
TEM images of an isolated PVDF/CNT fiber (**a**). The enlarged TEM image (**b**): indicating the CNTs embedded in the fiber. **c** The FTIR spectra of twisted electrospun PVDF/CNT ropes with MWCNTs of 8, 12.4, and 16.7 wt%

The TEM images in Fig. 7a, b show the CNTs with a diameter of 5–10 nm embedded in the PVDF matrix, which has proved the existence of the nanotubes in the as-prepared PVDF/CNT composite fibers. Additionally, the FTIR spectra (as shown in Fig. 7c) of twisted electrospun PVDF/CNT nanoropes with MWCNTs of 8, 12.4, and 16.7 wt% were processed in the wave number range of 700–1000 cm^−1^. It is obvious that there are characteristic absorption peaks of the PVDF/CNT nanocomposite β-phase at 840 cm^−1^ in each twisted rope. Moreover, the band of β-phase at 840 cm^−1^ is enhanced with the increasing content of MWCNTs, which agrees with the previous studies [40, 41].

### Mechanical and Electrical Properties of Aligned PVDF/CNT Bundles and Twisted Ropes

**Fig. 8:**
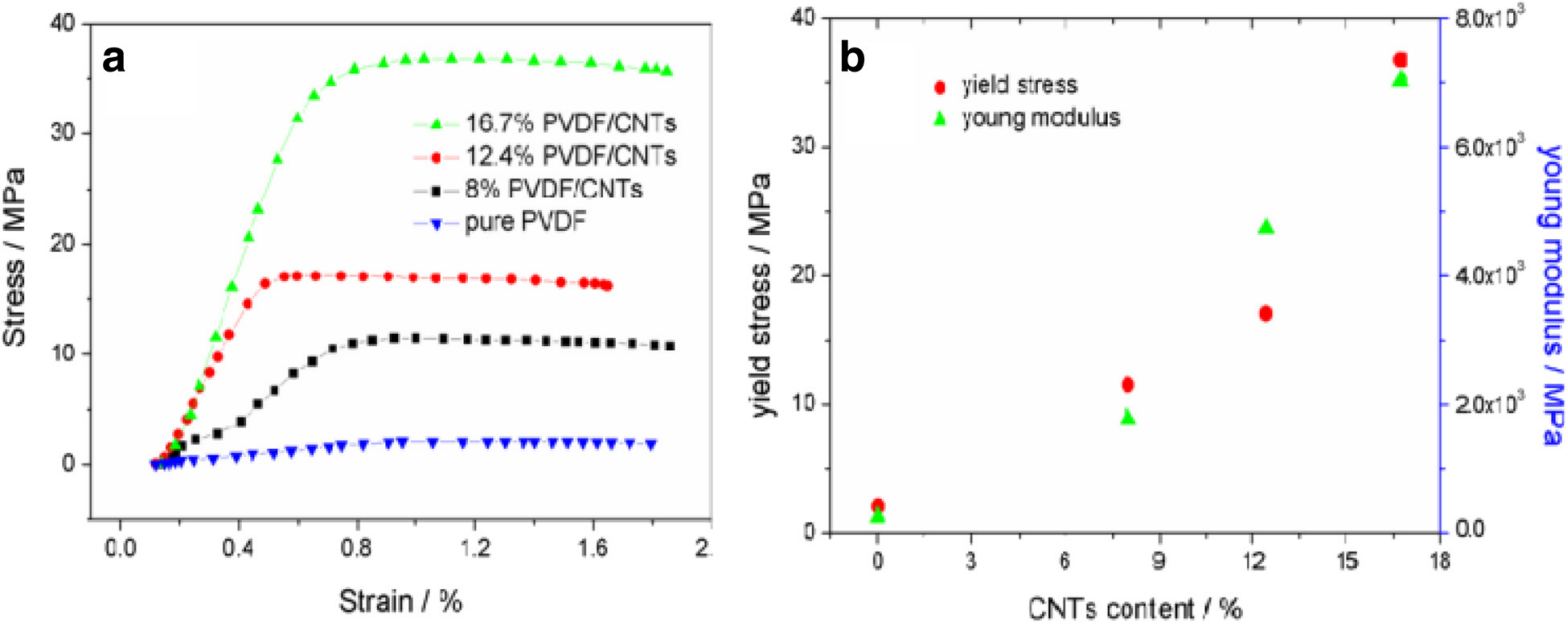
Mechanical properties of the PVDF/CNT twisted ropes with different CNT contents: **a** stress versus strain curves and **b** yield stress and the Young modulus

Compared to the loosely aligned PVDF/CNT composite fibers, twisted ropes show improved cohesion and friction between fibers and then exhibit good mechanical performance, as shown in Fig. 8. For the pure PVDF rope, the yield stress and Young modulus are only 2.05 MPa and 2.48 × 10^2^ MPa, respectively, while both of them increase by around one order of magnitude for PVDF/CNT rope with added 8 wt% CNTs (11.52 MPa and 1.78 × 10^3^ MPa, respectively). With the increase of the CNT content to 12.4 wt% in the composite ropes, the mechanical performance is improved continuously, leading to 17.05 MPa in yield stress and 4.74 × 10^3^ MPa in the Young modulus. When the CNT content increases up to 16.7 wt%, we obtain the highest values in yield stress and the Young modulus of 36.76 MPa and 7.02 × 10^3^ MPa, respectively. As a result, the mechanical performance of PVDF/CNT composite twisted ropes is improved significantly (the increase in yield stress and the Young modulus are around 20 and 30 times, respectively) compared with the pure PVDF twisted rope, resulting in the potential applications in nanodevices and biomimetics, such as artificial muscles, actuators, and nanoelectromechanical systems [11].

**Fig. 9:**
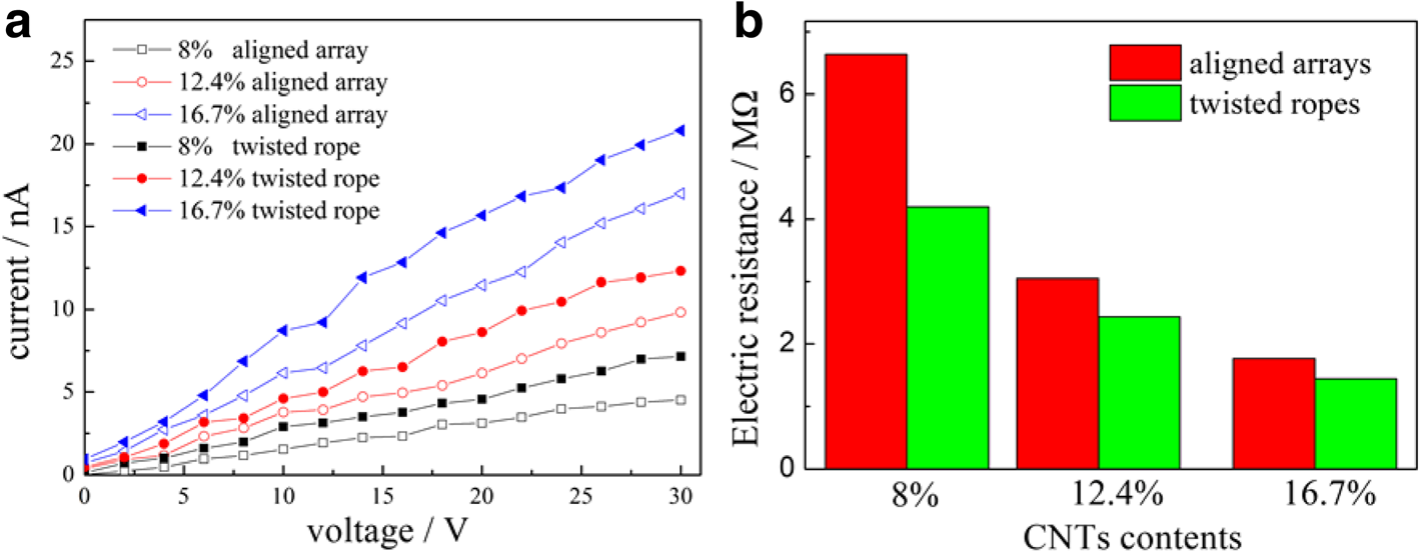
**a** I-V curves of the composite fiber bundles and twisted ropes. **b** Electric resistance of the six samples in (**a**)

The current-voltage (I-V) characteristics of the composite fiber arrays and twisted ropes are shown in Fig. 9a. Herein, all fibrous arrays and ropes were fabricated with the same experimental parameters. Their lengths are about 10 cm. The diameters are 75, 67, and 52 μm for the 8.0, 12.4, and 16.7 wt% twisted ropes, respectively, which are only one third to one half of the corresponding fiber bundles before twist due to more compact structure of the rope and elongation of the fibers. From the optical and SEM images in Figs. 3 and 6, it is obvious that the space between fibers of twisted ropes is much smaller leading to a stronger physical contact compared with fibrous array. This improved physical contact reduces the contact resistance between fibers of twisted ropes significantly, resulting in the conductivity increase in comparison with fibrous arrays (Fig. 9b). On the other hand, the conductivity mainly results from the addition of CNTs, so the CNT loading has a critical influence in the electrical properties. In both cases of aligned array and twisted rope, the electrical resistance decreases with increasing CNT content. The electrical resistance of the fiber array decreases from 6640, 3050, to 1780 MΩ, while the resistance of the corresponding twisted rope decreases from 4200, 2440, to 1400 MΩ, as shown in Fig. 9b. Twisted ropes have better electrical performance than fibrous arrays at the same CNT loading. The possible reason for this change may be due to improved fiber-fiber contacts from the fiber bundle to twisted rope.

### Strain Sensors Based on Twisted Ropes

**Fig. 10:**
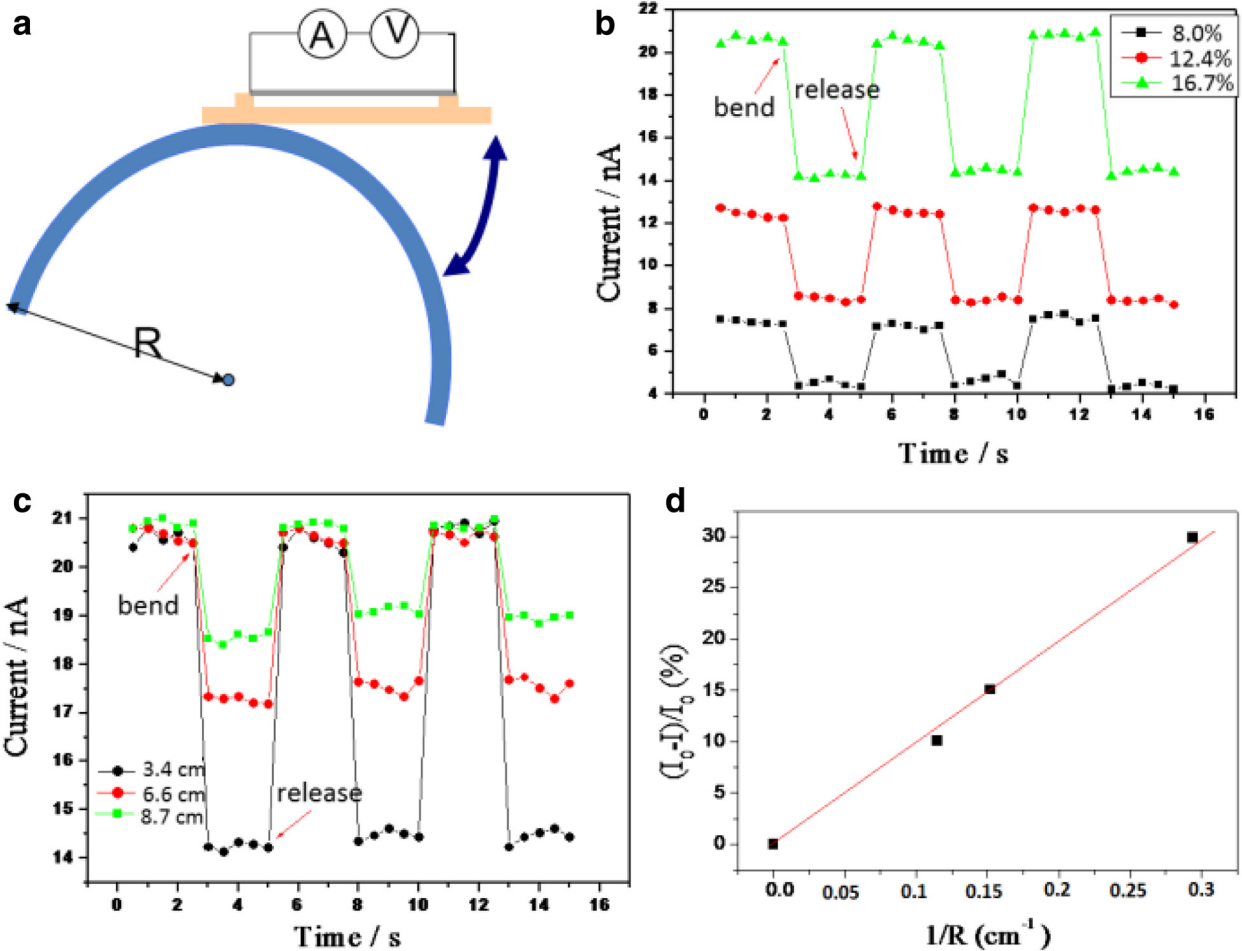
**a** Schematic diagram of the flexible strain sensor. **b** Current responses to bend and release the three sensors on a bending radius of 3.4 cm. **c** Current responses of the sensor (16.7 wt% PVDF/CNTs) on different bending radiuses of 3.4, 6.6, and 8.7 cm. **d** Calculated sensitivity (∆I/I_0_) of the 16.7 wt% PVDF/CNT sensor on different bending radiuses

Interestingly, these PVDF/CNT twisted ropes are sensitive to strain and can be used as high-strain sensors. The schematic diagram of the flexible sensor is shown in Fig. 10a. The twisted rope was fixed on a flexible insulating substrate (e.g., plastic film), and then, two thin copper wires were attached on the rope by silver paste as electrodes, which were then connected to a power supply and a current meter for electric measurement. When the device was bended onto a semicircle surface with a constant curvature, the current changed accordingly. The applied voltage was 30 V. From Fig. 10b, we can see the current decreased from 21 to 14 nA when the sensor (16.7 wt% PVDF/CNT twisted rope) was bended on a radius of 34 mm. After releasing the applied force, the current recovered to 21 nA within a second. With less CNT loadings such as 8 and 12.4 wt%, similar current responses are also observed. In addition, the high-strain sensors are very stable: the bending and releasing currents remain in the same level even after 20 cycles (only 3 cycles are shown in Fig. 10b). We also investigated the sensitivity of the 16.7 wt% PVDF/CNT sensor on different bending radiuses of 3.4, 6.6, and 8.7 cm. With the increase in curvature (1/R) or applied strain, the change of the current (∆I/I_0_) becomes larger, as shown in Fig. 10c, d. For instance, the sensitivity increases from 10.1 to 29.9 % when the bending radius decreases from 8.7 to 3.4 cm.

## Conclusions

In summary, a modified e-spinning setup with two-frame collector is introduced for fabrication of highly ordered arrays within an area up to 14 × 10 cm^2^. Based on the aligned arrays, twisted continuous ropes can be prepared. The results show that both the position of the inner frame and the rotation speed have significant influence on the morphologies of resultant twisted ropes. On the other hand, the mechanical properties of PVDF/CNT composite twisted ropes are improved by one order of magnitude, indicating their potential in many applications, such as artificial muscle and electronic devices. In addition, the strain-sensitive property of these composite twisted ropes indicates their potential use in strain sensors.

## References

[1] Huang ZM, Zhang YZ, Kotaki M, Ramakrishna S (2003). A review on polymer nanofibers by electrospinning and their applications in nanocomposites. Compos Sci Technol.

[2] Li MM, Yang DY, Long YZ, Ma HW (2010). Arranging junctions for nanofibers. Nanoscale.

[3] Li MM, Long YZ, Tan JS, Yin HX, Sui WM, Zhang ZM (2010). Dielectric properties of electrospun titanium compound/polymer composite nanofibres. Chin Phys B.

[4] Long YZ, Yu M, Sun B, Gu CZ, Fan ZY (2012). Recent advances in large-scale assembly of semiconducting inorganic nanowires and nanofibers for electronics, sensors and photovoltaics. Chem Soc Rev.

[5] Tan JS, Long YZ, Li MM (2008). Preparation of aligned polymer micro/nanofibres by electrospinning. Chin Phys Lett.

[6] Greiner A, Wendorff JH (2008). Functional self-assembled nanofibers by electrospinning. Adv Polym Sci.

[7] Reneker DH, Yarin AL (2008). Electrospinning jets and polymer nanofibers. Polymer.

[8] Schiffman JD, Schauer CL (2008). A review: electrospinning of biopolymer nanofibers and their applications. Polymer Rev.

[9] Agarwal S, Wendorff JH, Greiner A (2008). Use of electrospinning technique for biomedical applications. Polymer.

[10] Sundaray B, Subramanian V, Natarajan TS, Xiang RZ, Chang CC, Fann WS (2004). Electrospinning of continuous aligned polymer fibers. Appl Phys Lett.

[11] Gu BK, Sohn K, Kim SJ, Kim SI (2007). Fabrications of nanofibers as crossed arrays by electrospinning. J Nanosci Nanotechnol.

[12] Li MM, Long YZ, Yang DY, Sun JS, Yin HX, Zhao ZL, Kong WH, Jiang XY, Fan ZY (2011). Fabrication of one dimensional superfine polymer fibers by double-spinning. J Mater Chem.

[13] Sun DH, Chang C, Li S, Lin LW (2006). Near-field electrospinning. Nano Lett.

[14] Chang C, Limkrailassiri K, Lin LW (2008). Continuous near-field electrospinning for large area deposition of orderly nanofiber patterns. Appl Phys Lett.

[15] Rinaldi M, Ruggieri F, Lozzi L, Santucci S (2009). Well-aligned TiO_2_ nanofibers grown by near-field-electrospinning. J Vac Sci Technol B.

[16] Zheng GF, Li WW, Wang X, Wu DZ, Sun DH, Lin LW (2010). Precision deposition of a nanofibre by near-field electrospinning. J Phys D Appl Phys.

[17] Pu J, Yan XJ, Jiang YD, Chang C, Lin LW (2010). Piezoelectric actuation of direct-write electrospun fibers. Sensors Actuators A.

[18] Chang C, Tran VH, Wang JB, Fuh YK, Lin LW (2010). Direct-write piezoelectric polymeric nanogenerator with high energy conversion efficiency. Nano Lett.

[19] Katta P, Alessandro M, Ramsier RD, Chase GG (2004). Continuous electrospinning of aligned polymer nanofibers onto a wire drum collector. Nano Lett.

[20] Dalton PD, Klee D, Moller M (2008). Electrospinning with dual collection rings. Polymer.

[21] Gu BK, Shin MK, Sohn KW, Kim SI, Kim SJ (2007). Direct fabrication of twisted nanofibers by electrospinning. Appl Phys Lett.

[22] Liu LQ, Eder M, Burgert I, Tasis D, Prato M, Wagner HD (2007). One step electrospun nanofiber-based composite ropes. Appl Phys Lett.

[23] Maheshwari S, Chang HC (2009). Assembly of multi-stranded nanofiber threads through AC electrospinning. Adv Mater.

[24] Hansen BJ, Liu Y, Yang RS, Wang ZL (2010). Hybrid nanogenerator for concurrently harvesting biomechanical and biochemical energy. ACS Nano.

[25] Xiao X, Yuan LY, Zhong JW, Ding TP, Liu Y, Cai ZX, Rong YG, Han HW, Zhou J, Wang ZL (2011). High-strain sensors based on ZnO nanowire/polystyrene hybridized flexible films. Adv Mater.

[26] Lotus AF, Bender ET, Evans EA, Ramsier RD, Reneker DH, Chase GG (2008). Electrical, structural, and chemical properties of semiconducting metal oxide nanofiber yarns. J Appl Phys.

[27] Lotus AF, Bhargava S, Bender ET, Evans EA, Ramsier RD, Reneker DH, Chase GG (2009). Electrospinning route for the fabrication of p-n junction using nanofiber yarns. J Appl Phys.

[28] Lin DP, He HW, Huang YY, Han WP, Yu GF, Yan X, Long YZ, Xia LH (2014). Twisted microropes for stretchable devices based on electrospun conducting polymer fibers doped with ionic liquid. J Mater Chem C.

[29] Chen GX, Li YJ, Shimizu H (2007). Ultrahigh-shear processing for the preparation of polymer/carbon nanotube composites. Carbon.

[30] Owens FJ, Jayakody JRP, Greenbaum SGC (2006). Characterization of single walled carbon nanotube: polyvinylene difluoride composites. Compos Sci Technol.

[31] Huang S, Yee WA, Tjiu WC, Liu Y, Kotaki M, Yin CFB, Jan MA, Liu TX, Lu XH (2008). Electrospinning of polyvinylidene difluoride with carbon nanotubes: synergistic effects of extensional force and interfacial interaction on crystalline structures. Langmuir.

[32] Wang M, Shi JH, Pramoda KP, Goh SH (2007). Microstructure, crystallization and dynamic mechanical behaviour of poly(vinylidene fluoride) composites containing poly(methyl methacrylate)-grafted multiwalled carbon nanotubes. Nanotechnology.

[33] Baughman RH, Zakhidov AA, de Heer WA (2002). Carbon nanotubes—the route toward applications. Science.

[34] Collins PG, Arnold MS, Avouris P (2001). Engineering carbon nanotubes and nanotube circuits using electrical breakdown. Science.

[35] Zhang QH, Lippits DR, Rastogi S (2006). Dispersion and rheological aspects of SWNTs in ultrahigh molecular weight polyethylene. Macromolecules.

[36] Zhou CF, Liu T, Wang T, Kumar S (2006). PAN/SAN/SWNT ternary composite: pore size control and electrochemical supercapacitor behavior. Polymer.

[37] Qian D, Dickey EC, Andrews R, Rantell T (2000). Load transfer and deformation mechanisms in carbon nanotube-polystyrene composites. Appl Phy Lett.

[38] Meng Q, Wang K, Guo W, Fang J, Wei Z, She XL (2014). Thread‐like supercapacitors based on one‐step spun nanocomposite yarns. Small.

[39] Lima MD, Hussain MW, Spinks GM, Naficy S, Hagenasr D, Bykova JS, Tolly D and Baughman RH (2015) Efficient, absorption-powered artificial muscles based on carbon nanotube hybrid yarns. Small. doi: 10.1002/smll.201500424. 10.1002/smll.201500424 25755113

[40] Sarvi A, Chimello V, Silva AB, Bretas RES, Sundararaj U (2014). Coaxial electrospun nanofibers of poly (vinylidene fluoride)/polyaniline filled with multi-walled carbon nanotubes. Polymer Compos.

[41] Georgousis G, Pandis C, Kalamiotis A, Georgiopoulos P, Kyritsis A, Kontou E, Omastova M (2015). Strain sensing in polymer/carbon nanotube composites by electrical resistance measurement. Compos Part B: Eng.

